# Cachexia index for prognostication in surgical patients with locally advanced oesophageal or gastric cancer: multicentre cohort study

**DOI:** 10.1093/bjs/znae098

**Published:** 2024-04-09

**Authors:** Leo R Brown, Georgina G Thomson, Ellen Gardner, Siobhan Chien, Josh McGovern, Ross D Dolan, Stephen T McSorley, Matthew J Forshaw, Donald C McMillan, Stephen J Wigmore, Andrew B Crumley, Richard J E Skipworth

**Affiliations:** Clinical Surgery, University of Edinburgh, Royal Infirmary of Edinburgh, Edinburgh, UK; Department of General Surgery, Forth Valley Royal Hospital, Larbert, UK; Clinical Surgery, University of Edinburgh, Royal Infirmary of Edinburgh, Edinburgh, UK; Department of General Surgery, Forth Valley Royal Hospital, Larbert, UK; Centre for Sustainable Delivery, Golden Jubilee Hospital, Glasgow, UK; Academic Unit of Surgery, University of Glasgow, Glasgow Royal Infirmary, Glasgow, UK; Academic Unit of Surgery, University of Glasgow, Glasgow Royal Infirmary, Glasgow, UK; Academic Unit of Surgery, University of Glasgow, Glasgow Royal Infirmary, Glasgow, UK; Department of Upper Gastrointestinal Surgery, Glasgow Royal Infirmary, Glasgow, UK; Academic Unit of Surgery, University of Glasgow, Glasgow Royal Infirmary, Glasgow, UK; Clinical Surgery, University of Edinburgh, Royal Infirmary of Edinburgh, Edinburgh, UK; Department of General Surgery, Forth Valley Royal Hospital, Larbert, UK; Academic Unit of Surgery, University of Glasgow, Glasgow Royal Infirmary, Glasgow, UK; Clinical Surgery, University of Edinburgh, Royal Infirmary of Edinburgh, Edinburgh, UK

## Abstract

**Background:**

Features of cancer cachexia adversely influence patient outcomes, yet few currently inform clinical decision-making. This study assessed the value of the cachexia index (CXI), a novel prognostic marker, in patients for whom neoadjuvant chemotherapy and surgery for oesophagogastric cancer is planned.

**Methods:**

Consecutive patients newly diagnosed with locally advanced (T3–4 or at least N1) oesophagogastric cancer between 1 January 2010 and 31 December 2015 were identified through the West of Scotland and South-East Scotland Cancer Networks. CXI was calculated as (L3 skeletal muscle index) × (serum albumin)/(neutrophil lymphocyte ratio). Sex-stratified cut-off values were determined based on the area under the curve (AUC), and patients were divided into groups with low or normal CXI. Primary outcomes were disease progression during neoadjuvant chemotherapy and overall survival (at least 5 years of follow-up).

**Results:**

Overall, 385 patients (72% men, median age 66 years) were treated with neoadjuvant chemotherapy for oesophageal (274) or gastric (111) cancer across the study interval. Although patients with a low CXI (men: CXI below 52 (AUC 0.707); women: CXI below 41 (AUC 0.759)) were older with more co-morbidity, disease characteristics were comparable to those in patients with a normal CXI. Rates of disease progression during neoadjuvant chemotherapy, leading to inoperability, were higher in patients with a low CXI (28 *versus* 12%; adjusted OR 3.07, 95% c.i. 1.67 to 5.64; *P* < 0.001). Low CXI was associated with worsened postoperative mortality (*P* = 0.019) and decreased overall survival (median 14.9 *versus* 56.9 months; adjusted HR 1.85, 1.42 to 2.42; *P* < 0.001).

**Conclusion:**

CXI is associated with disease progression, worse postoperative mortality, and overall survival, and could improve prognostication and decision-making in patients with locally advanced oesophagogastric cancer.

## Teaser

Cachexia index represents a valuable marker of ‘host stage’, and a potential adjunct to routine tumour staging. This could improve prognostication and decision-making in patients with locally advanced oesophagogastric cancer.

## Introduction

Oesophagogastric (OG) cancer accounts for less than 5% of all malignancies diagnosed annually in Scotland, yet ranks as the third leading cause of cancer-related mortality^1,2^. It predominantly affects older patients, who often have more co-morbidity and are frail. Almost half of patients diagnosed with OG cancer have disseminated malignancy at the point of initial clinical staging^3^. As such, most patients only undergo treatment with palliative or best supportive intent. Outcomes for those on curative pathways have improved in recent years, driven by evolving operative and perioperative techniques, improved use of effective (neo)adjuvant oncological treatments, and involvement of specialist allied health professionals, as standard, throughout preoperative and postoperative care. Despite these advances, compared with other cancer types, long-term survival for OG cancer remains poor^3^. Tumour stage dictates the choice of management pathway for most patients with OG cancer. Age, performance status, and co-morbidities may preclude suitability for particular treatments, and are considered nominally.

Cancer cachexia is highly prevalent in patients with OG cancer^4^. Although cachexia is most often seen in patients with advanced stages of malignancy, it can also influence survival adversely in patients undergoing surgical resection for OG cancer^5^. Despite this, phenotypic features of cachexia are rarely discussed during multidisciplinary team (MDT) treatment planning within the current staging paradigm. Consideration of the extratumoral effects of cancer (‘host stage’) may be a valuable adjunct to tumour stage for risk stratification and identification of patients who are less likely to have a favourable outcome. The cachexia index (CXI) has been developed as a composite marker that incorporates several features of the cachectic phenotype, including low muscularity and systemic inflammatory response (SIR)^6^. These components are well aligned with the phenotypic and aetiological diagnostic criteria required by the Global Leadership Initiative on Malnutrition (GLIM) guidelines^7^, and are known to have prognostic value in isolation^8,9^. Although a small number of studies have demonstrated the adverse influence of a low CXI in various cancer sites and stages, the marker remains underexplored in both patients with OG cancer and in Western populations.

The aim of this study was to assess the prognostic value of CXI in patients undergoing neoadjuvant chemotherapy (NAC) before planned curative resection for locally advanced OG cancer.

## Methods

Data were collected for consecutive patients with a new diagnosis of oesophageal or gastric cancer between 1 January 2010 and 31 December 2015, allowing a follow-up of at least 5 years. Patients from six regional health boards were identified from the prospectively maintained databases of West of Scotland and South-East Scotland Cancer Networks. Together, these networks oversee almost three-quarters of all new cases of OG cancer in Scotland. Additional relevant variables were sought by retrospective interrogation of electronic patient records. Local approvals from National Health Service (NHS) Trust Caldicott Guardians were obtained before study commencement. This study was reported in line with STROBE guidance^10^.

### Patients

Included patients were those with locally advanced (T3–4) or node-positive (at least N1) disease, who received NAC with a plan for subsequent curative surgical resection. Patients who had neoadjuvant radiotherapy, underwent radical systemic anticancer treatment, had metastatic disease on clinical staging, and those who were treated with palliative intent were not eligible. Any patients who did not have satisfactory radiological imaging for body composition analysis or were missing other data required to calculate the CXI (serum albumin, neutrophil or lymphocyte count, or height) were also excluded.

### Clinical staging and management

All patients underwent diagnostic oesophagogastroduodenoscopy and thoracoabdominal CT as part of clinical staging. Staging was reported in accordance with the TNM classification (8th edition) then categorized retrospectively into AJCC clinical stage groups^11,12^. Histological subtypes were classed as adenocarcinoma or squamous cell carcinoma. Tumours of the gastro-oesophageal junction (GOJ) were classified according to Siewert based on findings at endoscopy. Type I and II GOJ tumours were considered oesophageal and type III GOJ tumours gastric. Almost all patients with oesophageal cancer (98%) also underwent PET–CT, but such imaging was used less frequently in patients with gastric cancer (27%). Conversely, staging laparoscopy and peritoneal washing-based cytology was undertaken more frequently in patients with gastric cancer (89%) than those with oesophageal cancer (54%). All patients were discussed at a specialist MDT meeting at the time of diagnosis (before NAC) and before surgery (after NAC).

Chemotherapy regimens varied over the study according to recruiting trials and the contemporary standard of care. The most frequently used regimens were cisplatin + 5-fluorouracil, or combinations of epirubicin with cisplatin + 5-fluorouracil (ECF); cisplatin + capecitabine (ECX); or oxaliplatin + capecitabine (EOX). All patients were restaged by thoracoabdominal CT following the completion of neoadjuvant treatment. Patients with oesophageal cancer underwent surgical resection comprising Ivor Lewis, left thoracoabdominal, transhiatal or three-stage oesophagectomy based on the tumour site and surgeon’s preference. Patients with gastric cancer had either a total or subtotal gastrectomy. Resections were performed routinely at two tertiary teaching hospitals (Glasgow Royal Infirmary and Royal Infirmary of Edinburgh) under the care of a subspecialist oesophagogastric surgeon.

### CT body composition analysis

Contrast-enhanced portal venous-phase CT images were analysed for each included patient at both staging (before NAC) and post-NAC (preoperative) time points. The cross-sectional areas (cm^2^) of skeletal muscle, visceral adipose tissue, and subcutaneous adipose tissue were measured at the midpoint of the third lumbar vertebrae (L3) level. The cross-sectional area of muscle, at the mid-L3 vertebral level, was normalized for height squared (m^2^) to create the skeletal muscle index (SMI) (cm^2^/m^2^). Skeletal muscle density (SMD) was calculated as the mean muscle radiodensity in Hounsfield units (HU) across the same region of interest. Body composition analyses were undertaken using Data Analysis Facilitation Suite (DAFS) (Voronoi Health Analytics, Vancouver, BC, Canada). This software performs automated tissue segmentation using non-linear image-processing algorithms, rather than identification based on predefined ranges of radiodensity for specific tissue types. The high levels of accuracy achieved by these algorithms have been validated previously against manual segmentation^13^.

### Cachexia index

Haematological and biochemical results were reviewed at staging and post-NAC time points. Neutrophil lymphocyte ratio (NLR) was calculated by dividing the absolute neutrophil count by the absolute lymphocyte count. CXI^6^ was calculated as follows:


$$\mathrm{CXI}=\;\frac{\mathrm{SMI}\;x\;\mathrm{Alb}}{\mathrm{NLR}}$$

where Alb is the serum albumin concentration measured in g/dl.

### Other definitions

ASA fitness grade and Charlson Co-morbidity Index score were collected as markers of co-morbidity. Eastern Cooperative Oncology Group (ECOG) score was reviewed as measure of performance status. Cut-offs were aligned with those featured in the GLIM criteria for low BMI (below 20 kg/m^2^ for age less than 70 years, or below 22 kg/m^2^ for age 70 years and over) and involuntary weight loss (over 5% in 3 months or less, or over 10% in 6 months or more). Radiologically diagnosed low muscularity was defined in accordance with Martin *et al*.^14^ (men: SMI less than 43 cm^2^/m^2^ if BMI below 25 kg/m^2^, SMI less than 53 cm^2^/m^2^ if BMI 25 kg/m^2^ or more; women: SMI less than 41 cm^2^/m^2^). NLR values were categorized as below 3, 3–5, and more than 5^15^, and albumin level was grouped as below 35 or 35 g/l and over, in line with thresholds from the modified Glasgow Prognostic Score^16^.

### Outcomes

Primary outcomes were disease progression during NAC (before surgical resection) and overall survival. All included patients had a minimum of 5 years’ follow-up from the date of starting neoadjuvant treatment. Secondary outcomes included 30-day postoperative mortality, postoperative duration of hospital stay, and complication rate. Complications were graded according to the Clavien–Dindo classification, and considered major if they had a grade of III or higher.

### Statistical analysis

Receiver operating characteristic (ROC) curve analyses were used to evaluate the classification ability of CXI. Optimal threshold binary values were defined by maximization of the Youden index for overall survival at 1 year from the date of starting neoadjuvant treatment. This cut-off was selected as a survival time that would be equivalent to that of patients with similar-stage disease who did not undergo curative therapy, owing to either preference or clinician-identified contraindication. The area under the curve (AUC) was computed with a 95% confidence interval based on 2000 stratified bootstrap replicates. Statistical comparison of AUC was based on the bootstrap percentile method. Characteristics and outcomes for patients with low *versus* normal CXI were compared. Continuous data were summarized as mean(s.d.) or median (i.q.r.) based on visual and statistical evaluation for normality. Subsequent testing was undertaken using appropriate parametric or non-parametric tests. Categorical data were cross-tabulated, and χ^2^ or Fisher’s exact test was used to assess differences. Kaplan–Meier analysis and curves were used to estimate survival, and groups were compared using the log rank test. Regression analyses were undertaken for primary outcomes of interest using logistic and Cox proportional hazards modelling, as appropriate. Clinically plausible confounders (age, sex, ASA grade, ECOG performance status, smoking status, tumour site, histology, clinical stage, grade, and BMI) were explored in preliminary models and first-order interactions were checked. Those selected for final models were based on minimization of the Akaike information criterion and maximization of the c-statistic. Explanatory variables with missing data were addressed using multiple imputation by chained equations to generate 10 multiply imputed data sets, which were pooled using Rubin’s rules. Data analysis was performed using R 4.2.2 (R Foundation for Statistical Computing, Vienna, Austria) with tidyverse, finalfit, pROC, mice, finalpsm, survival, and survminer packages.

## Results

Overall, 455 patients were identified. Data points necessary for calculation of the CXI were missing for 60 patients, most commonly height (88.3%). A further 10 patients were lost to follow-up having left the region within the 5-year minimum follow-up. After these exclusions, a final study cohort of 385 patients was available for subsequent analyses. All patients were treated with NAC for oesophageal (274) or gastric (111) cancer. The median age across the cohort was 66 (i.q.r. 59–71) years and 277 patients (71.9%) were men. BMI varied considerably across this patient group (median 26.1 (range 14.9–50.9) kg/m^2^). Data regarding involuntary pretreatment weight loss were available for 69.4% of patients. Across this subgroup, median weight loss was 3.2 (i.q.r. 0–6.4) kg or 4.5 (0–9.1)%. Weight loss was greater in patients with oesophageal than gastric cancer (*P* = 0.038), whereas other clinical characteristics were comparable between tumour sites (*Appendix S1*). Adenocarcinoma was the most common histological subtype of oesophageal cancer (238, 86.9%). Most patients had clinical stage III disease (290, 75.3%) with the disease graded as poorly differentiated (203, 64.4%) (*Appendix S2*).

### Influence of neoadjuvant chemotherapy on body composition and systemic inflammation

NAC commenced a median of 7 (i.q.r. 6–9) weeks following diagnosis. Most patients were treated with ECF, ECX, or EOF (244, 63.4%) but cisplatin + 5-fluorouracil (139, 36.1%) was also commonly used. Two patients received other regimens. Both SMI and SMD decreased during NAC administration (median SMI 47.8 *versus* 43.3 cm^2^/m^2^; median SMD 39.8 *versus* 38.4 HU; both *P* < 0.001) (*Appendix S3*). Cross-sectional areas of visceral and subcutaneous fat were also smaller after NAC (both *P* < 0.001). A decrease in NLR (2.6 *versus* 2.2; *P* < 0.001) was evident following NAC. CXI was comparable across both time points (staging: median 67.1 (i.q.r. 42.7–95.8); post-NAC: median 66.1 (42.8–92.1); *P* = 0.730).

### Cachexia index

**Fig. 1:**
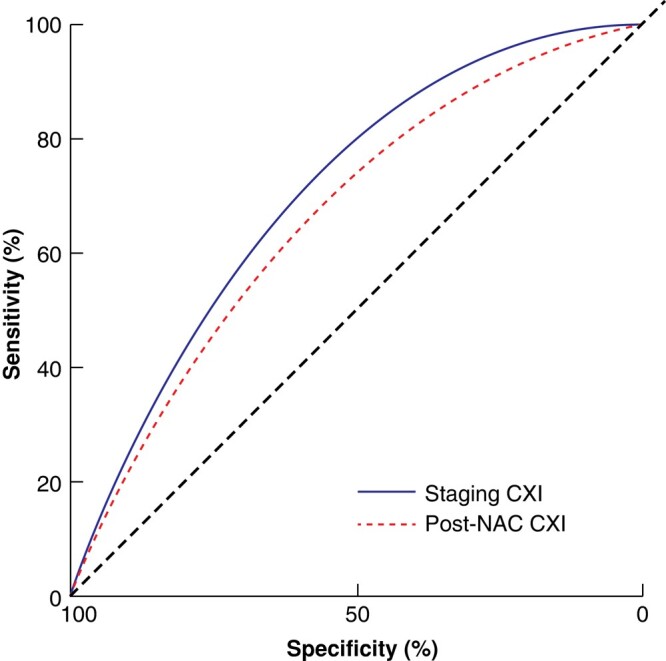
Smoothed receiver operating characteristic (ROC) curve comparison of staging cachexia index and postneoadjuvant chemotherapy cachexia index CXI, cachexia index; NAC, neoadjuvant chemotherapy. *P* = 0.509.

At the time of clinical staging, the median CXI was 74.5 (i.q.r. 50.6–106.3) for men and 51.3 (34.9–73.6) for women. Based on post-NAC investigation findings, the median CXI was 69.1 (44.5–101.1) for men and 59.5 (40.0–80.8) for women. The classification ability of staging CXI was comparable to that of post-NAC CXI, with an AUC of 0.721 (95% c.i. 0.648 to 0.793) *versus* 0.685 (0.600 to 0.770) respectively (*P* = 0.509) (*Fig. 1*). As progression during NAC was a co-primary endpoint for the present study, staging CXI offered greater utility and was therefore the focus of subsequent analyses. Sex-stratified optimum binary threshold values for a low CXI were below 52 for men (AUC 0.707, 95% c.i. 0.617 to 0.798; specificity 65.3%, sensitivity 80.3%) and below 41 for women (AUC 0.759, 0.659 to 0.860; specificity 84.2%, sensitivity 73.0%). Based on these cut-offs, 118 patients (30.6%) had a low CXI.

### Comparison of clinicopathological characteristics by staging cachexia index

Patients with a low CXI were older with more co-morbidity, according to ASA grade and Charlson Co-morbidity Index score (*Table 1*). The proportion of patients with an ECOG performance status score of 0 was smaller in the group with low CXI. The low-CXI group had lower bodyweight and albumin level, and higher NLR. Although similar rates of low CXI were seen across tumour sites, grades, and clinical stages, a greater proportion of patients with squamous cell carcinoma of the oesophagus more likely to have a low CXI (47.2%) than those with oesophagogastric adenocarcinoma (28.9%).

**Table 1:** Clinicopathological characteristics at staging by cachexia index

	Low CXI (*n* = 118)	Normal CXI (*n* = 267)	*P*‡
**Age (years), median (i.q.r.)**	68 (63–73)	64 (57–70)	< 0.001§
**Sex (M:F)**	78:40	199:68	0.109
**ASA fitness grade**			0.004
I	41 (34.7)	131 (49.1)	
II	43 (36.4)	98 (36.7)	
III	31 (26.3)	34 (12.7)	
IV	3 (2.5)	4 (1.5)	
**Charlson Co-morbidity Index score**			0.044
0–1	62 (52.5)	159 (59.6)	
2–4	51 (43.2)	106 (39.7)	
≥ 5	5 (4.2)	2 (0.7)	
**ECOG performance status score**			< 0.001
0	67 (56.8)	206 (77.2)	
1	42 (35.6)	50 (18.7)	
2	9 (7.6)	10 (3.7)	
3	0 (0)	1 (0.4)	
**Smoking status**			0.218
Smoker	19 (16.1)	58 (21.7)	
Ex-smoker	48 (40.7)	112 (41.9)	
Non-smoker	50 (42.4)	89 (33.3)	
Missing	1 (0.8)	8 (3.0)	
**Height (m), median (i.q.r.)**	1.7 (1.6–1.8)	1.7 (1.6–1.8)	0.184§
**Weight (kg), median (i.q.r.)**	70 (61.8–82.0)	76.8 (66.0–89.1)	< 0.001§
Missing	5 (4.2)	27 (10.1)	
**Tumour site**			0.821
Oesophagus	70 (59.8)	143 (53.8)	
GOJ (type I)	8 (6.8)	21 (7.9)	
GOJ (type II)	9 (7.7)	22 (8.3)	
GOJ (type III)	5 (4.3)	10 (3.8)	
Stomach	25 (21.4)	70 (26.3)	
**Histology**			0.035
AC	101 (85.6)	248 (92.9)	
SCC	17 (14.4)	19 (7.1)	
Clinical TNM stage*			0.190
II	14 (11.9)	40 (15.0)	
III	82 (69.5)	195 (73.0)	
IV	22 (18.6)	32 (12.0)	
**Differentiation (tumour grade)**			0.318
Poor (G3)	60 (50.9)	143 (53.6)	
Moderate (G2)	26 (22.0)	81 (30.3)	
Well (G1)	0 (0)	5 (1.9)	
Missing	32 (27.1)	38 (14.2)	
**Albumin (g/l)**, **median (i.q.r.)**	35 (33–38)	38 (35–40)	< 0.001§
< 35	49 (41.5)	45 (16.9)	< 0.001
≥ 35	69 (58.5)	222 (83.1)	
**Neutrophil lymphocyte ratio, median (i.q.r.)**	4.5 (3.6–5.7)	2.2 (1.7–2.7)	< 0.001§
< 3	7 (5.9)	234 (87.6)	< 0.001
3–5	62 (52.6)	33 (12.4)	
> 5	49 (41.5)	0 (0)	
**GLIM phenotypic criteria**†			
Weight loss			
Weight loss (kg), median (i.q.r.)	4.0 (0–9.0)	3.0 (0–6.4)	0.033§
Weight loss (%), median (i.q.r.)	5.8 (0–11.4)	3.3 (0–8.4)	0.027§
Losing weight	49 (41.5)	75 (28.1)	0.067
Stable weight	38 (32.2)	95 (35.6)	
Weight change missing	31 (26.3)	97 (36.3)	
BMI (kg/m^2^), median (i.q.r.)	25.1 (22.0–28.0)	26.6 (24.0–30.0)	< 0.001§
Low BMI	21 (17.8)	24 (9.0)	0.027
Normal/high BMI	92 (78.0)	216 (80.9)	
Missing	5 (4.2)	27 (10.1)	
SMI (cm^2^/m^2^), median (i.q.r.)	42.8 (37.5–48.3)	49.3 (43.0–55.0)	< 0.001§
Low muscularity	85 (72.0)	111 (41.6)	< 0.001
Normal muscularity	33 (28.0)	156 (58.4)	

Values are *n* (%) unless otherwise stated. *Clinical TNM staging with AJCC stage groups. †Cut-offs for weight loss (over 5% in 3 months or less, or over 10% in 6 months or more) and low BMI were in accordance with those described in the Global Leadership Initiative on Malnutrition criteria. Low muscularity was defined according to Martin *et al*.^14^. CXI, cachexia index; ECOG, Eastern Cooperative Oncology Group; GOJ, gastro-oesophageal junction; AC, adenocarcinoma; SCC, squamous cell carcinoma; SMI, skeletal muscle index. ‡χ^2^ or Fisher’s exact test, except §Mann–Whitney *U* test.

### Disease progression before surgical resection

Following NAC, 35 patients (7.5%) were found to have radiological evidence of progressive disease, no longer amenable to curative surgical resection. A further 30 patients (7.8%) had an open/close laparotomy owing to intraoperative identification of unresectable malignancy. Rates of disease progression during NAC were higher in patients with a low CXI (28 *versus* 12%; *P* < 0.001). After imputation and adjustment for confounders, low CXI remained a predictor of inoperable disease after NAC (adjusted OR 3.07, 95% c.i. 1.67 to 5.64; *P* < 0.001) alongside being a current smoker (adjusted OR 2.23, 1.05 to 4.74; *P* = 0.038) (*Table 2*). Median survival among patients who experienced progression during neoadjuvant chemotherapy was 11.2 (95% c.i. 9.8 to 13.7) months.

**Table 2:** Logistic regression model for disease progression before surgical resection

	Operable (*n* = 320)	Inoperable (*n* = 65)	Univariable analysis		Complete-case multivariable analysis		Imputed multivariable analysis	
			OR*	*P*	OR*	*P*	OR*	*P*
**Age (years), median (i.q.r.)**	66 (59–71)	66 (58–70)	0.99 (0.96, 1.02)	0.611				
**Sex**								
Male	232 (83.8)	45 (16.2)	1.00 (reference)					
Female	88 (81.5)	20 (18.5)	1.17 (0.64, 2.07)	0.593				
**ASA fitness grade**								
I	147 (85.5)	25 (14.5)	1.00 (reference)		1.00 (reference)		1.00 (reference)	
II	120 (85.1)	21 (14.9)	1.03 (0.54, 1.93)	0.929	0.85 (0.44, 1.64)	0.629	0.88 (0.46, 1.69)	0.699
III	48 (73.8)	17 (26.2)	2.08 (1.03, 4.16)	0.039	1.35 (0.62, 2.84)	0.438	1.46 (0.69, 3.07)	0.321
III	5 (71.4)	2 (28.6)	2.35 (0.32, 11.59)	0.322	1.53 (0.19, 8.66)	0.646	1.70 (0.27, 10.55)	0.569
**ECOG performance status score**								
0	228 (83.5)	45 (16.5)	1.00 (reference)					
1	76 (82.6)	16 (17.4)	1.07 (0.56, 1.96)	0.840				
2	15 (78.9)	4 (21.1)	1.35 (0.37, 3.93)	0.608				
3	1 (100)	0 (0)	–	–				
**Smoking status**								
Non-smoker	117 (84.2)	22 (15.8)	1.00 (reference)		1.00 (reference)		1.00 (reference)	
Ex-smoker	136 (85.0)	24 (15.0)	0.94 (0.50, 1.77)	0.843	0.94 (0.48, 1.84)	0.862	0.97 (0.50, 1.87)	0.924
Smoker	58 (75.3)	19 (24.7)	1.74 (0.87, 3.48)	0.115	2.15 (1.00, 4.69)	0.051	2.23 (1.05, 4.74)	0.038
**Tumour site**								
Oesophagus	231 (84.3)	43 (15.7)	1.00 (reference)		1.00 (reference)		1.00 (reference)	
Stomach	89 (80.2)	22 (19.8)	1.33 (0.74, 2.33)	0.329	1.68 (0.86, 3.25)	0.125	1.80 (0.93, 3.49)	0.082
**Histology**								
AC	290 (83.1)	59 (16.9)	1.00 (reference)					
SCC	30 (83.3)	6 (16.7)	0.98 (0.36, 2.32)	0.971				
**Clinical TNM stage**								
II	47 (87.0)	7 (13.0)	1.00 (reference)		1.00 (reference)		1.00 (reference)	
III	227 (81.9)	50 (18.1)	1.48 (0.67, 3.75)	0.367	2.57 (1.01, 7.42)	0.061	2.46 (0.91, 6.65)	0.075
IV	46 (85.2)	8 (14.8)	1.17 (0.39, 3.58)	0.781	1.70 (0.47, 6.33)	0.417	1.76 (0.50, 6.22)	0.380
**Differentiation**								
Poor	195 (96.1)	8 (3.9)	1.00 (reference)					
Moderate	103 (96.3)	4 (3.7)	0.95 (0.25, 3.08)	0.930				
Well	5 (100)	0 (0)	–	–				
**BMI (kg/m^2^), median (i.q.r.)**	26.1 (23.3–29.4)	26.2 (22.0–28.9)	0.98 (0.93, 1.03)	0.419	1.01 (0.95, 1.07)	0.757	1.01 (0.95, 1.07)	0.701
**Cachexia index**								
Normal	235 (88.0)	32 (12.0)	1.00 (reference)		1.00 (reference)		1.00 (reference)	
Low	85 (72.0)	33 (28.0)	2.85 (1.65, 4.94)	< 0.001	2.89 (1.57, 5.38)	0.001	3.07 (1.67, 5.64)	< 0.001

Values are *n* (%) unless otherwise stated; *values in parentheses are 95% confidence intervals. ECOG, Eastern Cooperative Oncology Group; AC, adenocarcinoma; SCC, squamous cell carcinoma.

A secondary logistic regression model was constructed in which components of the CXI (albumin, SMI, and NLR) were considered as separate variables (*Appendix S4*). Involuntary weight loss was also included as an alternative clinical marker of cachexia. Neither low radiological muscularity (adjusted OR 0.97, 0.52 to 1.79; *P* = 0.917) or albumin level (adjusted OR 1.17, 0.61 to 2.22; *P* = 0.632) alone was associated with disease progression during NAC. SIR, demonstrated by a NLR of at least 5, was strongly associated with inoperable disease after NAC (adjusted OR 4.04, 1.82 to 8.99; *P* = 0.001).

### Postoperative outcomes

A total of 320 patients proceeded to surgical resection with curative intent at a median time between completion of neoadjuvant treatment and surgery of 5 (i.q.r. 4–7) weeks (low CXI 85, normal CXI 235). Overall complication rates were 70.6% in the low-CXI group and 65.5% in the normal-CXI group (*Table 3*). The proportions of patients who experienced a major postoperative complication were comparable. The 30-day postoperative mortality rate was higher in patients with a low CXI (4.7 *versus* 0.4%; *P* = 0.019).

**Table 3:** Postoperative outcomes by cachexia index

	Low CXI (*n* = 85)	Normal CXI (*n* = 235)	P*
**Duration of hospital stay (days), median (i.q.r.)**	14 (11–18)	14 (10–18)	0.652†
**Resection margin**			0.137
R0	52 (61.2)	165 (70.2)	
R1	33 (38.8)	70 (29.8)	
**Postoperative complications (Clavien–Dindo grade)**			0.047
0	25 (29.4)	81 (34.5)	
I	7 (8.2)	22 (9.4)	
II	32 (37.6)	71 (30.2)	
III	11 (12.9)	40 (17.0)	
IV	5 (5.9)	20 (8.5)	
V	5 (5.9)	1 (0.4)	
**Major postoperative complication**			0.885
Yes	21 (24.7)	61 (26.0)	
No	64 (75.3)	174 (74.0)	
**30-day postoperative mortality**			0.019
Yes	4 (4.7)	1 (0.4)	
No	81 (95.3)	234 (99.6)	

Values are *n* (%) unless otherwise stated. Major postoperative complications were classified as those with a Clavien–Dindo grade of at least III. CXI, cachexia index. *χ^2^ or Fisher’s exact test, except †Mann–Whitney *U* test.

### Overall survival

**Fig. 2:**
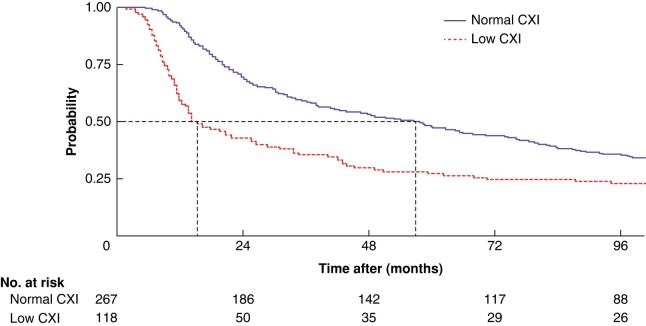
Kaplan–Meier overall survival analysis according to cachexia index group CXI, cachexia index. Dotted lines indicate median survival times. *P* < 0.001 (log rank test).

Median overall survival was 38.4 (95% c.i. 31.4 to 50.2) months. Survival was significantly reduced in the low-CXI cohort compared with that in the normal-CXI cohort: median 14.9 (12.6 to 27.4) *versus* 56.9 (41.2 to 73.9) months respectively (*P* < 0.001) (*Fig. 2*). The adverse survival associated with low CXI was evident at 1 year (59.3 *versus* 92.5%; *P* < 0.001), 3 years (35.6 *versus* 58.8%; *P* < 0.001) and 5 years (27.1 *versus* 47.9%; *P* < 0.001). This association was also noted among the subgroup of 320 patients who proceeded to surgical resection (low CXI: median 34.1 (20.6 to 59.1) months; normal CXI: median 70.1 (57.7 to 83.9) months; *P* = 0.007). In multivariable Cox regression, low CXI (adjusted HR 1.85, 95% c.i. 1.42 to 2.42; *P* < 0.001) was associated with poorer overall survival, after adjustment for relevant confounding variables (*Table 4*).

**Table 4:** Multivariable Cox proportional hazards model for overall survival

	All patients	Univariable analysis		Complete-case multivariable analysis		Imputed multivariable analysis	
		HR*	*P*	HR*	*P*	HR*	*P*
**Age (years), median (i.q.r.)**	66 (59– 71)	1.01 (1.00, 1.03)	0.049				
**Sex**							
Male	277 (71.9)	1.00 (reference)		1.00 (reference)		1.00 (reference)	
Female	108 (28.1)	0.68 (0.51, 0.90)	0.007	0.73 (0.51, 1.03)	0.073	0.69 (0.51, 0.92)	0.013
**ASA fitness grade**							
I	172 (44.7)	1.00 (reference)		1.00 (reference)		1.00 (reference)	
II	141 (36.6)	1.40 (1.07, 1.83)	0.013	1.30 (0.94, 1.78)	0.110	1.40 (1.07, 1.83)	0.016
III	65 (16.9)	1.65 (1.18, 2.30)	0.003	1.15 (0.75, 1.75)	0.528	1.46 (1.03, 2.07)	0.033
IV	7 (1.8)	2.50 (1.10, 5.69)	0.029	3.63 (1.28, 10.24)	0.015	2.73 (1.13, 6.59)	0.026
**ECOG performance status score**							
0	273 (70.9)	1.00 (reference)					
1	92 (23.9)	1.13 (0.85, 1.49)	0.398				
2	19 (4.9)	1.75 (1.06, 2.87)	0.028				
3	1 (0.3)	–	–				
**Smoking status**							
Non-smoker	139 (37.0)	1.00 (reference)		1.00 (reference)		1.00 (reference)	
Ex-smoker	160 (42.6)	0.86 (0.65, 1.12)	0.266	0.82 (0.59, 1.13)	0.225	0.87 (0.66, 1.15)	0.332
Smoker	77 (20.5)	1.08 (0.78, 1.49)	0.657	0.97 (0.64, 1.47)	0.882	1.15 (0.81, 1.63)	0.431
**Tumour site**							
Oesophagus	274 (71.2)	1.00 (reference)					
Stomach	111 (28.8)	0.94 (0.72, 1.22)	0.638				
**Histology**							
AC	349 (90.6)	1.00 (reference)					
SCC	36 (9.4)	0.87 (0.57, 1.32)	0.498				
**Clinical TNM stage**							
II	54 (14.0)	1.00 (reference)		1.00 (reference)		1.00 (reference)	
III	277 (71.9)	1.69 (1.15, 2.48)	0.008	1.89 (1.19, 3.02)	0.007	1.74 (1.16, 2.59)	0.007
IV	54 (14.0)	1.60 (0.98, 2.59)	0.059	1.54 (0.85, 2.80)	0.152	1.55 (0.93, 2.56)	0.091
**Differentiation (grade)**							
Poor (G3)	203 (64.4)	1.00 (reference)		1.00 (reference)		1.00 (reference)	
Moderate (G2)	107 (34.0)	0.65 (0.49, 0.88)	0.004	0.70 (0.51, 0.96)	0.028	0.70 (0.50, 0.98)	0.036
Well (G1)	5 (1.6)	0.34 (0.08, 1.37)	0.129	0.23 (0.03, 1.69)	0.151	0.34 (0.08, 1.43)	0.142
BMI (kg/m^2^), median i.q.r.)	26.1 (23.2–29.4)	0.98 (0.96, 1.01)	0.226	1.00 (0.97, 1.03)	0.899	0.99 (0.96, 1.02)	0.453
**Cachexia index**							
Normal	267 (69.4)	1.00 (reference)		1.00 (reference)		1.00 (reference)	
Low	118 (30.6)	1.84 (1.43, 2.36)	< 0.001	1.65 (1.19, 2.29)	0.003	1.85 (1.42, 2.42)	< 0.001

Values are *n* (%) unless otherwise stated; *values in parentheses are 95% confidence intervals. ECOG, Eastern Cooperative Oncology Group; AC, adenocarcinoma; SCC, squamous cell carcinoma.

A secondary Cox regression model was constructed in which components of the CXI (albumin, SMI, and NLR) were considered separately, alongside involuntary weight loss (*Appendix S5*). Although an adverse effect of radiologically diagnosed low muscularity was evident on univariable analysis (HR 1.32, 1.04 to 1.68; *P* = 0.021), following imputation and adjustment for other variables, this effect was no longer evident (adjusted HR 1.25, 0.96 to 1.63; *P* = 0.096). Low albumin level was not associated with poorer survival in univariable or multivariable analyses. After adjustment for confounding variables, NLR 3–5 (adjusted HR 1.72, 1.29 to 2.31; *P* < 0.001) and NLR over 5 (adjusted HR 1.64, 1.13 to 2.38; *P* = 0.009) were associated with worse overall survival. Staging NLR and CXI were subsequently compared as binary classifiers using ROC curve analysis (*Appendix S6*). Although no significant difference was evident between the two markers (*P* = 0.747), the AUC was greater for staging CXI than staging NLR: 0.721 (0.648 to 0.793) *versus* 0.716 (0.645 to 0.786).

### Subgroup analysis by tumour stage and site

Subgroup analysis by clinical tumour stage identified a survival advantage associated with a normal CXI in patients with cTNM stage III and IV disease (*Appendix S7*). Worse overall survival was evident in patients with oesophageal cancer but not among those with gastric cancer (*Appendix S8*).

## Discussion

This study has identified an adverse prognostic effect associated with a low CXI in patients with locally advanced OG cancer. Median overall survival in this group was 3.5 years shorter than that of patients with a normal CXI. This negative effect remained evident after adjustment for known confounders. Low CXI was also associated with progression to unresectable disease during NAC and higher rates of postoperative mortality, despite equivalent major postoperative complication rates. Low CXI may therefore represent poorer physiological reserve required to overcome incident complications. When contributing features of the CXI were analysed in isolation, the most influential component was the SIR. These findings highlight the potential value of cachectic markers, such as those included within the CXI, for informing shared decision-making in this high-risk cohort.

Contemporary survival data still show that approximately 50% of patients who undergo ‘curative’ OG cancer resection die within 5 years^3^. Survival in the present selected cohort of patients with locally advanced OG cancer was unsurprisingly even lower. Prognosis is inextricably linked to disease stage. Patients with T3–4 or at least N1 OG cancer are a particularly borderline cohort, in whom disease progression may occur during NAC, and long-term survival is achieved in only a small proportion of those treated with curative intent. Furthermore, the oncological and surgical treatments undertaken in pursuit of cure are known to be associated with considerable morbidity and mortality. Improved risk stratification may help clinicians counsel patients more effectively regarding treatment options and prognosis. Although recent, advanced attempts at survival prediction for resected OG cancer^17^ have shown excellent discrimination, these models require data points that would only be known after completion of a patient’s initial treatment (for example, postoperative complications and resection pathology staging). As such, their use is restricted to prognostication in the postoperative follow-up setting. Furthermore, these models do not include known valuable markers of the extratumoral effects of cancer. The influence of cachexia remains understudied in OG cancer, particularly among curative treatment cohorts.

Jafri *et al*.^6^ first devised the CXI as a composite marker that combined multiple clinical features of the cachectic phenotype. The group highlighted decreased overall and progression-free survival in patients with metastatic non-small cell lung cancer and a low CXI. Similarly adverse survival outcomes have since been shown for a number of other cancer sites. Previous analyses by Gong *et al*.^18^ and Sakurai *et al.*^19^ evaluated CXI in cohorts of patients who underwent surgical resection of gastric cancer, and both identified low CXI as an adverse predictor of survival. The present study has shown that this negative effect is also evident in oesophageal cancer, and in cohorts treated with NAC before planned surgical resection. This selected cohort of patients with locally advanced OG cancer is a very relevant group on which to focus this research question owing to the potential for cure yet often notably poor prognosis. Although it is unlikely that a low CXI could justify the choice of a non-curative treatment pathway in a fit and well patient with cTNM stage I disease, the decision is often far less clear in those with stage II or III malignancy. This patient group is one in which further risk stratification would be more clinically useful.

It is not surprising that a low CXI is associated with worse outcomes in patients with cancer. Previous investigation has consistently validated the adverse prognostic value of its components: radiological low muscularity^20^ and biochemical markers of SIR and/or poor nutrition^21^. However, the multidimensional pathophysiology of cachexia makes a composite marker or scoring system, which incorporates multiple phenotypic features, appealing. Several such assessment tools have been created in attempt to address this issue^22–24^, but complexity or need for non-routinely collected variables has largely precluded their clinical application. The methodology by which the CXI formula was derived was not reported by Jafri *et al*.^6^, but its simplicity suggests that the authors may not have assessed the optimum weighting that should be given to the marker’s components. The secondary regression models presented in *Appendices S4* and *S7* of the present work considered these components in isolation. No significant prognostic effect was identified for serum albumin level in this cohort. Although radiological low muscularity was associated with worse survival on univariable analysis, the effect was no longer evident following adjustment for other variables. Only NLR, as a marker of SIR, retained its prognostic value in multivariable analysis. This supports previous findings, such as that reported by Hacker *et al.*^25^ in an analysis of patients with OG cancer from the EXPAND trial, which suggested that SIR is the dominant prognostic host factor. Those authors proposed a causal link from SIR to sarcopenia, but no prognostic effect of body composition in the absence of an inflammatory response. The importance of inflammation in oesophagogastric cancer^9^ and cachexia^26^ has been increasingly acknowledged, and indeed GLIM guidelines feature it as one of two required aetiological diagnostic criteria^7^. However, no choice of inflammatory marker or cut-off values have been specified by GLIM, and what constitutes chronic disease-related inflammation remains relatively open to interpretation. Although having one of the three potential phenotypic criteria (weight loss, BMI, reduced muscle mass) may be sufficient for a diagnosis of disease-related malnutrition, it is not known whether these options are all equally applicable or important in particular cohorts, such as patients with cancer. Furthermore, other tissue measurements that can be obtained from CT body composition analyses, such as radiodensity and adiposity, may also have a prognostic effect^27^. These merit further exploration including longitudinal assessment^28^, and with other imaging modalities. Future efforts to develop a methodologically robust risk stratification tool or score should aim to use the most influential host markers.

Within the finite existing CXI literature, there are limitations across a number of key areas. First, the marker has been explored mostly in Asian populations. Given the significant geographical variations in body habitus, the cut-off values derived for a low CXI from these cohorts are unlikely to be comparable to those measured in Western populations. In a USA-based study^29^ that considered CXI, its prognostic influence was evaluated in cohorts of patients with advanced-stage malignancies only. These subgroups are likely to have sustained more aggressive or long-standing tissue wasting at the point of assessment and the data are therefore similarly at risk of being non-generalizable. Furthermore, variation even existed in methods for calculation of CXI in previous studies. A number of these did not use the L3 cross-sectional area of skeletal muscle to calculate SMI, but instead chose alternative measures based on psoas major^30^ or pectoralis^31^ muscles. These issues provided the rationale for determining optimized cut-offs specifically for the present study. However, as this research area develops further, consensus regarding what constitutes a low CXI should be sought.

The present study sought to provide long-term survival data on a large cohort of patients with locally advanced OG cancer. As a consequence, the authors are unable to comment on the influence of CXI in patients treated with more contemporary NAC regimens, such as FLOT (fluorouracil, leucovorin, oxaliplatin and docetaxel)^32^, for which long-term real-world data are not yet available. Ongoing tissue wasting has been shown to occur during both FLOT^33^ and pre-FLOT^34^ regimens of NAC in patients with OG cancer, but it is not yet known how regimens compare. Data regarding toxicities experienced during chemotherapy and dose adjustments were not captured prospectively, and could not be reliably identified from electronic patient records. As such, this should be acknowledged as a limitation of the present analysis. Additionally, the results may differ among cohorts treated using neoadjuvant chemoradiotherapy, such as that described in the CROSS trial^35^. Even now, this modality is used less frequently in the authors’ patient population in which adenocarcinoma predominates. Although investigative and management pathways were similar across the contributing centres, practice will have inevitably varied subtly between hospitals, clinicians, and over time. This includes the use of different CT scanners and scan protocol variations, which could theoretically influence body composition analyses. Heterogeneity in this cohort of oesophageal and gastric cancers, of both adenocarcinoma and squamous cell carcinoma subtypes, could be seen as either a limitation or strength of the study. This is demonstrative of the case diversity within the clinical practice of MDTs in Western populations. Future prospective validation of the CXI should be undertaken across a greater number of centres internationally. Such work would be helpful for further developing understanding of the potential influence of differing neoadjuvant treatment modalities or populations on the prognostic value of the CXI.

## Supplementary Material

znae098_Supplementary_Data

## Data Availability

Anonymized data may be available from the corresponding author upon reasonable request.
