# Supplementary material for: Cachexia index for prognostication in surgical patients with locally advanced oesophageal or gastric cancer: multicentre cohort study
Source: Br J Surg. 2024 Apr 9;111(4):znae098. doi: 10.1093/bjs/znae098 (PMC11003541; doi:10.1093/bjs/znae098)
Supplement: znae098_Supplementary_Data [file znae098_supplementary_data.docx]

**‘Cachexia Index’ for Prognostication in Surgical Patients with Locally Advanced Oesophageal or Gastric Cancer: A Multicentre Cohort Study**

Leo R. Brown ^1,2^, Georgina G. Thomson ^1^, Ellen Gardner ^2^, Siobhan Chien ^3^, Josh McGovern ^4^, Ross D. Dolan ^4^, Stephen T. McSorley ^4^, Matthew J. Forshaw ^5^, Donald C. McMillan ^4^, Stephen J. Wigmore ^1^, Andrew B. Crumley ^2,4*^, Richard J.E. Skipworth ^1*^

1. *Clinical Surgery, University of Edinburgh, Royal Infirmary of Edinburgh, Edinburgh, Scotland.*
2. *Department of General Surgery, Forth Valley Royal Hospital, Larbert, Scotland.*
3. *Golden Jubilee Hospital, Glasgow, Scotland.*
4. *Academic Unit of Surgery, University of Glasgow, Glasgow Royal Infirmary, Glasgow, Scotland.*
5. *Department of Upper GI Surgery, Glasgow Royal Infirmary, Glasgow, Scotland.*

*Joint Senior Authors

**Corresponding author:** Mr. Leo R. Brown, Clinical Surgery, University of Edinburgh, Royal Infirmary of Edinburgh, Edinburgh, Scotland. Email: leorbrown@doctors.org.uk **ORCID ID**: https://orcid.org/0000-0001-6181-7020

**Twitter:** @leorbrown

**Supplementary Materials - Index**

| **Supplementary Methods**  None |  |
| --- | --- |
| **Supplementary Results** |  |
| None |  |
| **Supplementary Appendixes** |  |
| None |  |
| **Supplementary Figures and Tables** |  |
| Appendix 1: Clinical Characteristics at Diagnosis by Tumour Site  Appendix 2: Pathological Characteristics at Clinical Staging by Tumour Site  Appendix 3: Changes in CT Body Composition and Systemic Inflammation with NAC | *pag. 3*  *pag. 4*  *pag. 5* |
| Appendix 4: Multivariable Logistic Regression Model for Disease Progression Prior to Surgical Resection (Model 2) | *pag. 6/7* |
| Appendix 5: Multivariable Cox Proportional Hazards Model for Overall Survival  (Model 2) | *pag. 8/9* |
| Appendix 6: Smoothed receiver operating characteristic (ROC) curve comparison of staging CXI (blue) and staging NLR (red) | *pag. 10* |
| Appendix 7: Kaplan Meier Survival Analysis (Low vs. Normal Cachexia Index) Stratified by cTNM Stage. | *pag. 11* |
| Appendix 8: Kaplan Meier Survival Analysis (Low vs. Normal Cachexia Index) Stratified by Tumour Site | *pag. 12* |
| **References**  None |  |
|  |  |

**Supplementary Figures and Tables**

**Appendix 1: Clinical Characteristics at Diagnosis by Tumour Site**

|  | | **Oesophageal**  **(n=274)** | **Gastric**  **(n=111)** | **p value** |
| --- | --- | --- | --- | --- |
| **Age** | Median [IQR] | 65 [59 to 70] | 68 [60.5 to 73] | 0.020 |
| **Sex** | Male | 205 (74.8) | 72 (64.9) | 0.060 |
|  | Female | 69 (25.2) | 39 (35.1) |  |
| **ASA Grade** | 1 | 130 (47.4) | 42 (37.8) | 0.371 |
|  | 2 | 95 (34.7) | 46 (41.4) |  |
|  | 3 | 44 (16.1) | 21 (18.9) |  |
|  | 4 | 5 (1.8) | 2 (1.8) |  |
| **Charlson** | 0-1 | 149 (54.4) | 72 (64.9) | 0.065 |
| **Comorbidity** | 2-4 | 118 (43.1) | 39 (35.1) |  |
| **Index** | ≥5 | 7 (2.6) | 0 (0.0) |  |
| **Height (m)** | Median [IQR] | 1.70 [1.60 to 1.80] | 1.70 [1.60 to 1.70] | 0.080 |
| **Weight** | Median [IQR] | 75.0 (65.7 to 88.1] | 72.0 [62.2 to 87.0] | 0.108 |
| **(kg)** | Missing | 30 (10.9) | 2 (1.8) |  |
| **BMI** | Median [IQR] | 26.1 [23.4 to 29.4] | 26.1 [22.3 to 29.2] | 0.601 |
| **(kg/m^2^)** | Missing | 30 (10.9) | 2 (1.8) |  |
| **Weight Loss** | Median [IQR] | 3.2 [0.0 to 7.0] | 2.0 [0.0 to 5.8] | 0.047 |
| **(kg)*** | Missing | 85 (31.0) | 33 (29.7) |  |
| **ECOG** | 0 | 194 (70.8) | 79 (71.2) | 0.970 |
|  | 1 | 66 (24.1) | 26 (23.4) |  |
|  | 2 | 13 (4.7) | 6 (5.4) |  |
|  | 3 | 1 (0.4) | 0 (0.0) |  |
| **Smoking** | Smoker | 54 (19.7) | 23 (20.7) | 0.672 |
| **Status** | Ex-smoker | 116 (42.3) | 44 (39.6) |  |
|  | Non-smoker | 96 (35.0) | 43 (38.7) |  |
|  | Missing | 8 (2.9) | 1 (0.9) |  |

Data are displayed as number (%) unless stated otherwise. ASA: American Society of Anaesthesiologists. BMI: Body Mass Index. ECOG: Eastern Cooperative Oncology Group performance status. *Patient reported involuntary weight loss during the preceding 6 months.

**Appendix 2: Pathological Characteristics at Clinical Staging by Tumour Site**

|  | | **Oesophageal**  **(n=274)** | **Gastric**  **(n=111)** | **p value** |
| --- | --- | --- | --- | --- |
| **Histological**  **Subtype** | Adenocarcinoma | 238 (86.9) | 111 (100.0) | N/A |
|  | Squamous Cell | 36 (13.1) | 0 (0.0) |  |
| **cT Stage** | 1 | 4 (1.5) | 0 (0.0) | <0.001 |
|  | 2 | 27 (9.9) | 19 (17.1) |  |
|  | 3 | 230 (83.9) | 73 (65.8) |  |
|  | 4 | 13 (4.7) | 19 (17.1) |  |
| **cN Stage** | 0 | 54 (19.7) | 23 (20.7) | 0.981 |
|  | 1 | 166 (60.6) | 66 (59.5) |  |
|  | 2 | 37 (13.5) | 16 (14.4) |  |
|  | 3 | 17 (6.2) | 6 (5.4) |  |
| **cTNM Stage**  **(AJCC)** | 2 | 14 (5.1) | 40 (36.0) | <0.001 |
|  | 3 | 210 (76.6) | 67 (60.4) |  |
|  | 4 | 50 (18.2) | 4 (3.6) |  |
| **Histological Grade**  **(Differentiation)** | Poor (G3) | 147 (53.6) | 56 (50.5) | 0.818 |
|  | Moderate (G2) | 76 (27.7) | 31 (27.9) |  |
|  | Well (G1) | 3 (1.1) | 2 (1.8) |  |
|  | Missing | 48 (17.5) | 22 (19.8) |  |

All data are displayed as number (%) unless stated otherwise. cTNM: Clinical Tumour (T) Node (N) and Metastasis (M) staging. American Joint Committee on Cancer (AJCC) stage groupings.

**Appendix 3: Changes in CT Body Composition and Systemic Inflammation with NAC**

1. **Overall**

|  | | **Staging** | **Post-NAC** | **p value** |
| --- | --- | --- | --- | --- |
| **SMI (cm^2^/m^2^)** | Median [IQR] | 47.8 [40.9 to 53.9] | 43.3 [37.1 to 49.3] | <0.001 |
| **SMD (HU)** | Median [IQR] | 39.8 [33.1 to 46.2] | 38.4 [32.2 to 44.4] | <0.001 |
| **VAT Area (cm^2^)** | Median [IQR] | 159.2 [78.1 to 229.7] | 140.4 [66.0 to 210.80] | <0.001 |
| **SAT Area (cm^2^)** | Median [IQR] | 162.7 [111.8 to 233.8] | 156.4 [98.9 to 219.5] | <0.001 |
| **NLR** | Median [IQR] | 2.6 [1.9 to 3.5] | 2.2 [1.6 to 3.3] | <0.001 |
| **Albumin (g/L)** | Median [IQR] | 37 [35 to 39] | 36 [32 to 38] | <0.001 |
| **CXI** | Median [IQR] | 67.1 [44.5 to 95.8] | 66.1 [42.8 to 92.1] | 0.730 |

1. **Male**

|  | | **Staging** | **Post-NAC** | **p value** |
| --- | --- | --- | --- | --- |
| **SMI (cm^2^/m^2^)** | Median [IQR] | 50.1 [45.6 to 55.7] | 46.3 [41.0 to 51.9] | <0.001 |
| **SMD (HU)** | Median [IQR] | 39.2 [32.9 to 45.7] | 37.7 [32.3 to 44.4] | 0.198 |
| **VAT Area (cm^2^)** | Median [IQR] | 177.1 [101.7 to 261.6] | 160.7 [98.2 to 229.8] | 0.104 |
| **SAT Area (cm^2^)** | Median [IQR] | 177.1 [115.1 to 238.5] | 159.4 [108.6 to 223.8] | 0.224 |
| **NLR** | Median [IQR] | 2.5 [1.8 to 3.4] | 2.2 [1.6 to 3.3] | 0.195 |
| **Albumin (g/L)** | Median [IQR] | 37 [35 to 39] | 36 [32 to 38] | <0.001 |
| **CXI** | Median [IQR] | 74.3 [50.6 to 109.8] | 70.2 [44.3 to 103.7] | 0.943 |

1. **Female**

|  | | **Staging** | **Post-NAC** | **p value** |
| --- | --- | --- | --- | --- |
| **SMI (cm^2^/m^2^)** | Median [IQR] | 38.6 [34.7 to 42.7] | 36.6 [32.7 to 40.5] | 0.001 |
| **SMD (HU)** | Median [IQR] | 40.5 [34.5 to 46.7] | 38.7 [32.0 to 44.1] | 0.075 |
| **VAT Area (cm^2^)** | Median [IQR] | 92.0 [44.0 to 180.2] | 62.8 [35.6 to 128.7] | 0.095 |
| **SAT Area (cm^2^)** | Median [IQR] | 139.7 [94.5 to 196.2] | 134.5 [88.7 to 208.8] | 0.916 |
| **NLR** | Median [IQR] | 2.8 [2.2 to 3.6] | 2.2 [1.7 to 3.1] | 0.037 |
| **Albumin (g/L)** | Median [IQR] | 37 [34 to 39.5] | 36 [32 to 38] | 0.021 |
| **CXI** | Median [IQR] | 50.9 [35.4 to 73.6] | 57.1 [41.3 to 79.9] | 0.027 |

SMI: Skeletal Muscle Index. SMD: Skeletal Muscle Density. HU: Hounsfield Units. VAT: Visceral Adipose Tissue. SAT: Subcutaneous Adipose Tissue. IMAT: Intramuscular Adipose Tissue. NLR: Neutrophil to Lymphocyte Ratio. CXI: Cachexia Index.

**Appendix 4: Multivariable Logistic Regression Model for Disease Progression Prior to Surgical Resection (Model 2)**

|  |  | Operable n=320 | Inoperable  n=65 | Univariable  OR (95% CI) | *p*  value | Complete Case  Multivariable  OR (95% CI) | *p*  value | Imputed  Multivariable  OR (95% CI) | *p* value |
| --- | --- | --- | --- | --- | --- | --- | --- | --- | --- |
| Age  (years) | Median  [IQR] | 66  [59 to 71] | 66  [58 to 70] | 0.99 (0.96-1.02) | 0.611 | - | - | - | - |
| Sex | Male | 232 (83.8) | 45 (16.2) | - | - | - | - | - | - |
|  | Female | 88 (81.5) | 20 (18.5) | 1.17 (0.64-2.07) | 0.593 | - | - | - | - |
| ASA | 1 | 147 (85.5) | 25 (14.5) | - | - | - | - | - | - |
|  | 2 | 120 (85.1) | 21 (14.9) | 1.03 (0.54-1.93) | 0.929 | 0.90 (0.41-1.98) | 0.797 | 0.95 (0.49-1.87) | 0.892 |
|  | 3 | 48 (73.8) | 17 (26.2) | 2.08 (1.03-4.16) | 0.039 | 1.76 (0.74-4.15) | 0.196 | 1.56 (0.73-3.31) | 0.249 |
|  | 4 | 5 (71.4) | 2 (28.6) | 2.35 (0.32-11.59) | 0.322 | 1.06 (0.05-9.10) | 0.962 | 1.99 (0.30-13.19) | 0.473 |
| ECOG | 0 | 228 (83.5) | 45 (16.5) | - | - | - | - | - | - |
|  | 1 | 76 (82.6) | 16 (17.4) | 1.07 (0.56-1.96) | 0.840 | - | - | - | - |
|  | 2 | 15 (78.9) | 4 (21.1) | 1.35 (0.37-3.93) | 0.608 | - | - | - | - |
|  | 3 | 1 (100.0) | 0 (0.0) | NA | NA | - | - | - | - |
| Smoking | Non-Smoker | 117 (84.2) | 22 (15.8) | - | - | - | - | - | - |
| Status | Ex-Smoker | 136 (85.0) | 24 (15.0) | 0.94 (0.50-1.77) | 0.843 | 0.96 (0.43-2.16) | 0.922 | 1.13 (0.57-2.24) | 0.732 |
|  | Smoker | 58 (75.3) | 19 (24.7) | 1.74 (0.87-3.48) | 0.115 | 2.37 (0.98-5.81) | 0.056 | 2.15 (0.99-4.64) | 0.053 |
| Tumour | Oesophagus | 231 (84.3) | 43 (15.7) | - | - | - | - | - | - |
| Site | Stomach | 89 (80.2) | 22 (19.8) | 1.33 (0.74-2.33) | 0.329 | 1.62 (0.73-3.55) | 0.232 | 1.96 (0.98-3.93) | 0.057 |
| Histology | AC | 290 (83.1) | 59 (16.9) | - | - | - | - | - | - |
|  | SCC | 30 (83.3) | 6 (16.7) | 0.98 (0.36-2.32) | 0.971 | - | - | - | - |
| cTNM | II | 47 (87.0) | 7 (13.0) | - | - | - | - | - | - |
| Stage | III | 227 (81.9) | 50 (18.1) | 1.48 (0.67-3.75) | 0.367 | 2.28 (0.79-7.59) | 0.149 | 2.29(0.84-6.25) | 0.104 |
|  | IV | 46 (85.2) | 8 (14.8) | 1.17 (0.39-3.58) | 0.781 | 1.02 (0.22-4.69) | 0.984 | 1.42 (0.38-5.27) | 0.599 |
| Grade | Poor | 195 (96.1) | 8 (3.9) | - | - | - | - | - | - |
|  | Moderate | 103 (96.3) | 4 (3.7) | 0.95 (0.25-3.08) | 0.930 | - | - | - | - |
|  | Well | 5 (100.0) | 0 (0.0) | NA | NA | - | - | - | - |
| BMI  (kg/m^2^) | Median  [IQR] | 26.0  [23.3 to 29.4] | 26.2  [22.0 to 28.9] | 0.98 (0.93-1.03) | 0.419 | 1.00 (0.94-1.06) | 0.970 | 1.00 (0.94-1.06) | 0.932 |
| Weight  Loss (kg) | Median  [IQR] | 3  [0 to 6.4] | 4  [0 to 10.5] | 1.06 (1.02-1.10) | 0.003 | 1.05 (1.00-1.10) | 0.044 | 1.05 (1.00-1.10) | 0.054 |
| SMI | Normal | 160 (50.0) | 29 (44.6) | - | - | - | - | - | - |
| (cm^2^/m^2^) | Low | 160 (50.0) | 36 (55.4) | 1.24 (0.73-2.13) | 0.429 | 0.89 (0.44-1.80) | 0.745 | 0.97 (0.52-1.79) | 0.917 |
| Albumin | ≥35 | 247 (77.2) | 44 (67.7) | - | - | - | - | - | - |
|  | <35 | 73 (22.8) | 21 (32.3) | 1.61 (0.89-2.86) | 0.106 | 1.24 (0.58-2.58) | 0.578 | 1.17 (0.61-2.22) | 0.632 |
| NLR | <3 | 213 (66.6) | 28 (43.1) | - | - | - | - | - | - |
|  | 3-5 | 75 (23.4) | 20 (30.8) | 2.03 (1.07-3.80) | 0.028 | 2.06 (0.91-4.60) | 0.079 | 1.95 (0.99-3.84) | 0.053 |
|  | >5 | 32 (10.0) | 17 (26.2) | 4.04 (1.97-8.18) | <0.001 | 3.17 (1.26-8.02) | 0.014 | 4.04 (1.82-8.99) | 0.001 |

Data are displayed as number (%) unless stated otherwise. OR: Odds Ratio, displayed with (95% confidence interval). ASA: American Society of Anaesthesiologists. ECOG: Eastern Cooperative Oncology Group performance status. BMI: Body Mass Index. *Patient reported involuntary weight loss during the preceding 6 months. AC: Adenocarcinoma. SCC: Squamous Cell Carcinoma. cTNM: Clinical TNM Staging with American Joint Committee on Cancer (AJCC) stage groupings. SMI: Skeletal Muscle Index. NLR: Neutrophil to Lymphocyte Ratio.

**Appendix 5: Multivariable Cox Proportional Hazards Model for Overall Survival (Model 2)**

|  |  | All n (%) | Univariable  HR (95% CI) | *p* value | Complete Case Multivariable  HR (95% CI) | *p*  value | Imputed  Multivariable  HR (95% CI) | *p* value |
| --- | --- | --- | --- | --- | --- | --- | --- | --- |
| Age | Median  [IQR] | 66  [59 to 71] | 1.01 (1.00-1.03) | 0.049 | - | - | - | - |
| Sex | Male | 277 (71.9) | - |  | - |  | - | - |
|  | Female | 108 (28.1) | 0.68 (0.51-0.90) | 0.007 | 0.67 (0.45-1.01) | 0.055 | 0.67 (0.50-0.90) | 0.009 |
| ASA | 1 | 172 (44.7) | - | - | - | - | - | - |
|  | 2 | 141 (36.6) | 1.40 (1.07-1.83) | 0.013 | 1.36 (0.92-1.99) | 0.122 | 1.49 (1.13-1.97) | 0.005 |
|  | 3 | 65 (16.9) | 1.65 (1.18-2.30) | 0.003 | 1.28 (0.76-2.13) | 0.351 | 1.60 (1.13-2.27) | 0.009 |
|  | 4 | 7 (1.8) | 2.50 (1.10-5.69) | 0.029 | 2.99 (0.89-10.06) | 0.077 | 3.25 (1.36-7.73) | 0.008 |
| ECOG | 0 | 273 (70.9) | - | - | - | - | - | - |
|  | 1 | 92 (23.9) | 1.13 (0.85-1.49) | 0.398 | - | - | - | - |
|  | 2 | 19 (4.9) | 1.75 (1.06-2.87) | 0.028 | - | - | - | - |
|  | 3 | 1 (0.3) | NA | NA | - | - | - | - |
| Smoking | Non-Smoker | 139 (37.0) | - | - | - | - | - | - |
| Status | Ex-Smoker | 160 (42.6) | 0.86 (0.65-1.12) | 0.266 | 0.71 (0.48-1.07) | 0.099 | 0.86 (0.65-1.15) | 0.309 |
|  | Smoker | 77 (20.5) | 1.08 (0.78-1.49) | 0.657 | 0.86 (0.52-1.41) | 0.551 | 1.05 (0.74-1.47) | 0.793 |
| Tumour | Oesophagus | 274 (71.2) | - | - | - | - | - | - |
| Site | Stomach | 111 (28.8) | 0.94 (0.72-1.22) | 0.638 | - | - | - | - |
| Histology | AC | 349 (90.6) | - | - | - | - | - | - |
|  | SCC | 36 (9.4) | 0.87 (0.57-1.32) | 0.498 | - | - | - | - |
| cTNM | II | 54 (14.0) | - | - | - | - | - | - |
| Stage | III | 277 (71.9) | 1.69 (1.15-2.48) | 0.008 | 1.39 (0.81-2.37) | 0.232 | 1.74 (1.17-2.61) | 0.007 |
|  | IV | 54 (14.0) | 1.60 (0.98-2.59) | 0.059 | 0.99 (0.50-1.97) | 0.983 | 1.54 (0.92-2.58) | 0.102 |
| Grade | Poor | 203 (64.4) | - | - | - | - | - | - |
|  | Moderate | 107 (34.0) | 0.65 (0.49-0.88) | 0.004 | 0.75 (0.52-1.09) | 0.130 | 0.69 (0.51-0.94) | 0.018 |
|  | Well | 5 (1.6) | 0.34 (0.08-1.37) | 0.129 | NA | NA | 0.50 (0.13-1.84) | 0.292 |
| BMI (kg/m^2^) | Median  [IQR] | 26.1  [23.2 to 29.4] | 0.98 (0.96-1.01) | 0.226 | 1.00 (0.97-1.04) | 0.908 | 0.98 (0.96-1.01) | 0.173 |
| Weight  Loss (kg) | Median  [IQR] | 3  [0 to 6.4] | 1.03 (1.01-1.05) | 0.001 | 1.02 (1.00-1.05) | 0.076 | 1.02 (1.00-1.05) | 0.075 |
| SMI | Normal | 189 (49.1) | - | - | - | - | - | - |
| (cm^2^/m^2^) | Low | 196 (50.9) | 1.32 (1.04-1.68) | 0.021 | 1.21 (0.84-1.74) | 0.310 | 1.25 (0.96-1.63) | 0.096 |
| Albumin | ≥35 | 291 (75.6) | - | - | - | - | - | - |
|  | <35 | 94 (24.4) | 1.17 (0.89-1.54) | 0.248 | 0.76 (0.50-1.16) | 0.207 | 0.95 (0.71-1.27) | 0.733 |
| NLR | <3 | 241 (62.6) | - | - | - | - |  |  |
|  | 3-5 | 95 (24.7) | 1.57 (1.19-2.06) | 0.001 | 1.80 (1.16-2.79) | 0.009 | 1.66 (1.29-2.31) | <0.001 |
|  | >5 | 49 (12.7) | 1.81 (1.28-2.57) | 0.001 | 1.16 (0.70-1.94) | 0.560 | 1.64 (1.13-2.38) | 0.009 |

HR: Hazard Ratio, displayed with 95% confidence interval. ASA: American Society of Anaesthesiologists. ECOG: Eastern Cooperative Oncology Group Performance Status. BMI: Body Mass Index. *Patient reported involuntary weight loss during the preceding 6 months. AC: Adenocarcinoma. SCC: Squamous Cell Carcinoma. cTNM: Clinical TNM Staging with American Joint Committee on Cancer (AJCC) stage groupings. SMI: Skeletal Muscle Index. NLR: Neutrophil to Lymphocyte Ratio.

**Appendix 6: Smoothed receiver operating characteristic (ROC) curve comparison of staging CXI (blue) and staging NLR (red)**

**
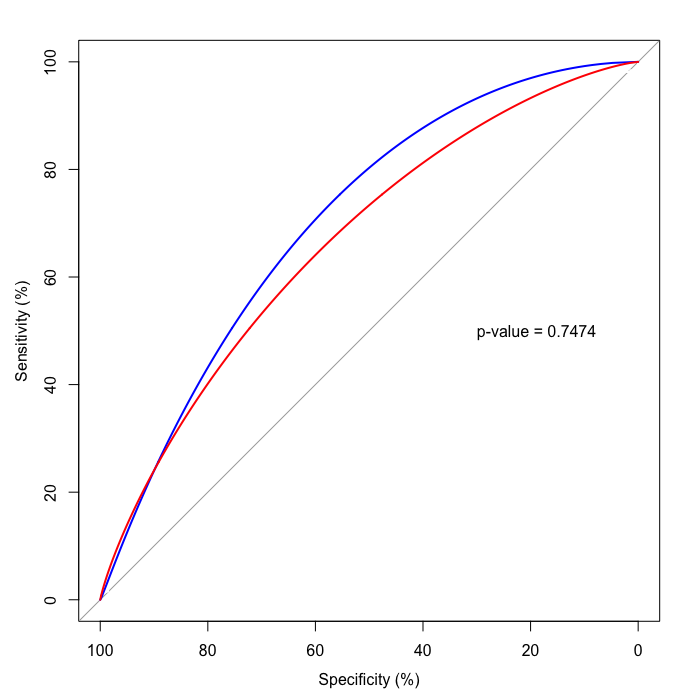
**

**Appendix 7: Kaplan Meier Survival Analysis (Low vs. Normal Cachexia Index) Stratified by cTNM Stage.**

| 1. **(A) Stage II** | **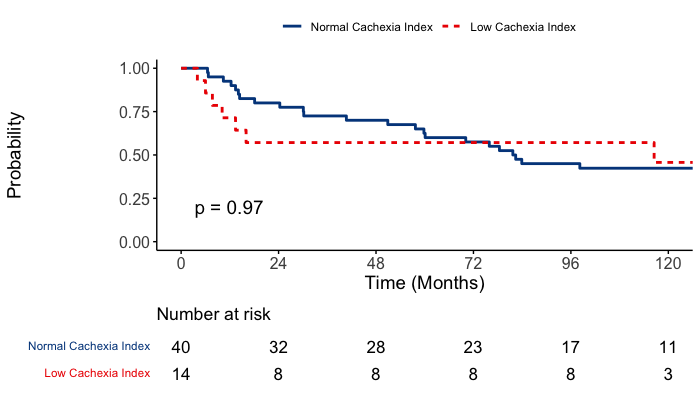** |
| --- | --- |
| 1. **(B) Stage III** | **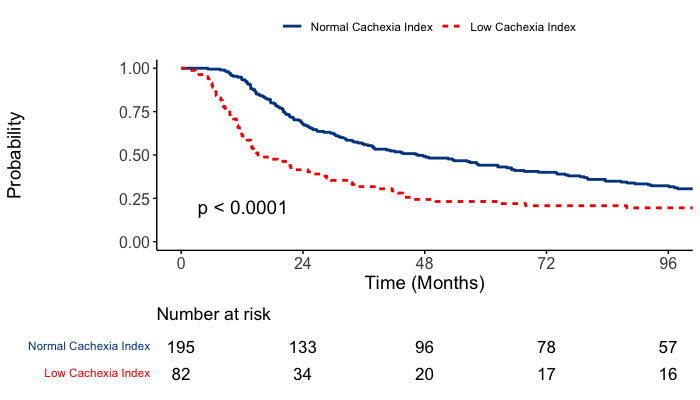** |
| 1. **(C) Stage IV** | **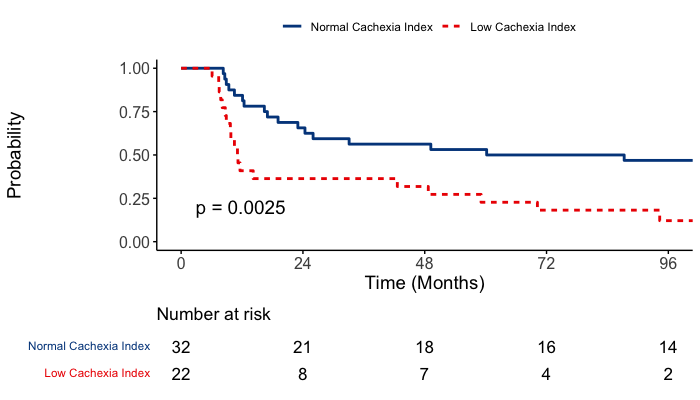** |

**Appendix 8: Kaplan Meier Survival Analysis (Low vs. Normal Cachexia Index) Stratified by Tumour Site**

| 1. **(A) Oesophageal** | **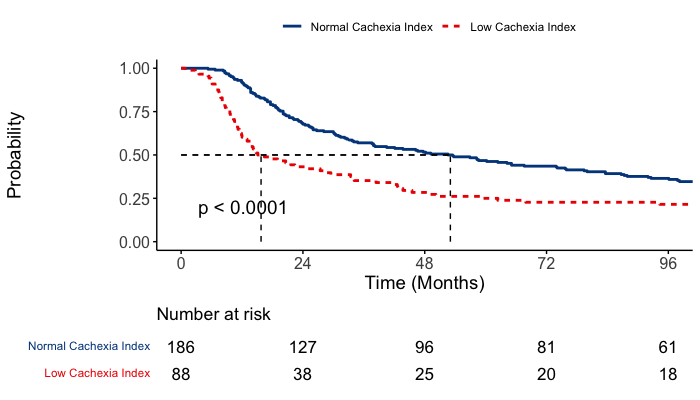** |
| --- | --- |
| 1. **(B) Gastric** | **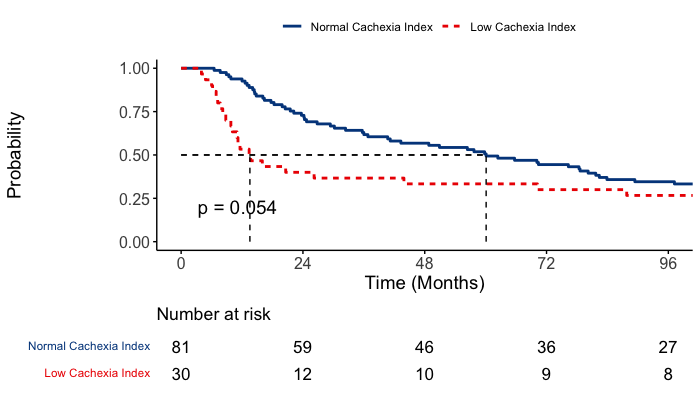** |
